# Experience in recruiting youths in HIV vaccine trials in Tanzania: the TaMoVac 01 study

**DOI:** 10.1186/1742-4690-9-S2-P125

**Published:** 2012-09-13

**Authors:** T Massawa, A Swalehe, D Niima

**Affiliations:** 1 Muhimbili University of Health and Allied Sciences, Dar es Salaam, Tanzania, United Republic of

## Background

Muhimbili University of Health Allied Science has been conducting HIV Vaccine trials since 2007. The first trial the HIVIS 03 study began in February 2007 that recruited Police. We believe that the population to be prevented from HIV would be youths. We describe our experice in recruiting youths.

## Methods

Sensitization meetings were conducted at the youth clinic.Pre screening workshops were conducted at Muhimbili National Hospital. Youths who showed interest in volunteering were asked to come for screening at the clinic located at Muhimbili National Hospital.

## Results

Enrolment of youth volunteers in the study was not a problem. Among 60 volunteers recruited 25 were youths. We recruited 5 males and 20 females who were recruited over 1 year. There were challenges encountered in recruiting youths. These included inability for independent decision to join the trail though we noted that the parents were supportive after being well informed. 4/25 youths relocated in search for jobs this resulted in additional costs to call in the volunteer for safety assessments. 2/25 female youths became pregnant during the study period.

## Conclusion

We noted that it was easy to recruit youths in the trial after the parents were well informed. However, there is need for continuous education so as to address pregnancy prevention.

